# A Case of Concomitant COVID-19 Infection-Induced Acute Respiratory Distress Syndrome and Diabetic Ketoacidosis: Another Challenge in Fluid Management

**DOI:** 10.7759/cureus.11805

**Published:** 2020-11-30

**Authors:** Panadeekarn Panjawatanan, Samir Jha, Joseph Hughes, Erik Riesenfeld

**Affiliations:** 1 Internal Medicine, Bassett Medical Center, Cooperstown, USA; 2 Department of Endocrinology, Bassett Medical Center, Cooperstown, USA; 3 Department of Pulmonary and Critical Care Medicine, Bassett Medical Center, Cooperstown, USA

**Keywords:** covid-19, acute respiratory distress syndrome [ards], diabetic ketoacidosis (dka)

## Abstract

Coronavirus disease 2019 (COVID-19) has been announced as a pandemic worldwide. The respiratory tract is a target organ system, where infection can result in serious complications, like acute respiratory distress syndrome (ARDS). Management of this condition is more challenging in individuals with diabetes who developed diabetic ketoacidosis. We report a case of a 59-year-old male with type 2 diabetes who presented with productive cough, chills, and shortness of breath for four days. On examination, the patient was hypoxemic with bilateral crackles on lung auscultation. The patient’s biochemistry was significant for glucose 387 mg/dL, pH 7.25, positive urine ketones, and lactic acid dehydrogenase (LDH) 325 U/L. An initial chest x-ray showed bilateral peripheral pulmonary infiltrates. The patient was subsequently intubated on the first day for worsening hypoxia due to severe ARDS. He was concomitantly treated for diabetic ketoacidosis (DKA) and hypotension with fluid resuscitation and intravenous insulin. On the same day, his hypoxia worsened with an increase in pulmonary infiltrates, so we stopped intravenous fluids and initiated norepinephrine for 24 hours. His intravenous insulin was initially started at 12 units/hour with subsequent titration down to an average of 5 units/hour. His mechanical ventilation settings followed ARDS guidelines with tidal volume 6 ml/kg based on ideal body weight. Positive COVID-19 was detected from real-time reverse transcription polymerase chain reaction (RT-PCR). After maintaining a negative fluid balance, we were able to extubate in 72 hours. DKA was resolved in 46 hours. In conclusion, type 2 diabetes is rarely affected by DKA, but can be found in up to 27% of cases. There are reports of ARDS as a serious complication in severe DKA in adults and children, yet no data for concomitant DKA and ARDS has been published. We propose that DKA management in COVID-19 patients with ARDS may be similar to the paradigm utilized for other volume restriction in patients with congestive heart failure and end-stage renal failure.

## Introduction

The first outbreak of severe acute respiratory syndrome coronavirus 2 (SARS-CoV-2) occurred in December 2019 in Wuhan, China. With its rapid rate of infection, Coronavirus disease 2019 (COVID-19) has been announced as a pandemic with more than 30 million infected worldwide; more than one-fifth of those affected reside in the US. An infected patient can either be a silent carrier or clinically symptomatic with fever, cough, sputum production, hemoptysis, dyspnea, sore throat, myalgia, headache, and/or gastrointestinal symptoms [1]. The pathogenesis of this infection is not yet clearly understood, although many hypothesize that it is related to cytokine release and systemic inflammation. The most commonly affected organ system is the respiratory tract, where infection can result in serious complications like acute respiratory distress syndrome (ARDS). ARDS occurs in 17.2-67.3% of cases according to a recent meta-analysis [1]. A variety of management modalities for ARDS include specific ventilator settings, prone positioning, and conservative volume management.

Among COVID-19 patients, 0.1-19.5% are found to have diabetes mellitus as an underlying disease [1]. This population is susceptible to severe infection due to their impaired immunity, the prevalence in the elderly, and association with cardiovascular disease [2]. One of the serious complications in diabetes is diabetic ketoacidosis (DKA), which can be triggered by infection, inadequate insulin therapy, pancreatitis, myocardial infarction, or medication. Dehydration is a common consequence due to hyperglycemia-induced glucosuria. Aggressive intravenous fluid resuscitation is important for volume repletion along with insulin therapy and electrolyte management [3].

Here we describe a patient with COVID-19 infection-induced ARDS with concurrent DKA who was managed with cautious volume resuscitation and vasopressors, a method drawn from a similar model of volume-sensitive patients in end-stage renal failure and congestive heart failure. He was successfully extubated within 72 hours of admission.

## Case presentation

We report a case of a 59-year-old male who presented with four days of productive cough with blood-tinged sputum, shortness of breath, and chills, along with some sinus congestion, sore throat, diarrhea, headache, and generalized body aches. The patient had decreased oral intake and had not been compliant with his diabetes medications. He had underlying disease significant for type 2 diabetes, essential hypertension, obesity (BMI 32 kg/m2), history of pancreatitis and diabetic ketoacidosis. His diabetes medications included insulin degludec 126 units with insulin lispro sliding scale, dulaglutide, metformin, and sitagliptin. He was a non-smoker and did not use vaping devices. The patient was working at one of the local intermediate care facilities one week prior to symptom development, and there was no history of exposure to COVID-19 positive patients or history of travel to a level 2 or 3 region. 

On examination, the patient was lethargic. Initial vital signs included a temperature of 36.8°C, respiratory rate 24/min, heart rate 65 bpm, blood pressure 140/67 mmHg, and oxygen saturation 91% on room air. Lung auscultation revealed bilateral widespread crackles. Laboratory testing was significant for hyperglycemia (glucose 387 mg/dL) with increased anion gap metabolic acidosis (corrected with albumin) and concurrent normal gap acidosis, positive urine ketones, elevated hemoglobin A1C, elevated lactic acid dehydrogenase (LDH), and troponin (demand ischemia) (Table 1). An initial chest x-ray showed bilateral peripheral pulmonary infiltrates. He was suspected to have COVID-19 infection. An infectious workup for influenza, rapid strep test, pneumococcal and legionella urinary antigen tests were negative. 

**Table 1:** Hematology and biochemistry profile on the first day of admission

	Result	Reference Value
White blood cell count (x10^3^ cells/uL)	9.6	3.7-10
Neutrophil (%)	84	40-70
Lymphocyte (%)	12	12-50
Platelet (x10^3^ cells/uL)	259	140-425
Glucose (mg/dL)	387	70-140
Urine ketones (mg/dL)	15	Negative
Hemoglobin A_1C_ (%)	11.3	4.0-5.5%
pH	7.25	7.35-7.48
pCO_2_ (mmHg)	44	32-48
pO_2_ (mmHg)	53	83-108
Sodium (mmol/L)	136	136-145
Potassium (mmol/L)	3.8	3.4 -4.5
Chloride (mmol/L)	104	98-110
Bicarbonate (mmol/L)	19	21-31
Blood urine nitrogen (mg/dL)	18	7-25
Creatinine (mg/dL)	0.9	0.7-1.3
Lactic acid (mmol/L)	1.1	0.5-2.2
Albumin (g/dL)	2.9	3.5-5.5
Lactate dehydrogenase (U/L)	325	140-271
Aspartate aminotransferase (U/L)	140	13-39
Alanine aminotransferase (U/L)	59	7-72
Alkaline phosphatase (U/L)	90	34-104
Bilirubin (mg/dL)	0.6	0.3-1
Amylase (U/L)	18	29-103
Lipase (U/L)	17	11-82
Troponin (high-sensitivity) (pg/mL)	943.8	<20

The patient was admitted due to worsening hypoxia. He developed fever and lymphopenia (absolute lymphocyte count 795 cells/uL, range 950-3,500 uL) during hospitalization. The patient subsequently required intubation and mechanical ventilation. His acute hypoxic respiratory failure was consistent with ARDS according to Berlin definition and was classified as severe ARDS (PaO2/FiO2 ratio of 71). The patient was concomitantly diagnosed with DKA due to hyperglycemia with elevated anion gap metabolic acidosis with positive urine ketone and normal lactate. Moreover, his clinical course was complicated by hypotension, likely multifactorial - DKA, sepsis, and sedative medication. He was treated with variable rate of intravenous insulin infusion, initially started at 12 units/hour with subsequent titration down to average of 5 units/hour, fluid resuscitation (approximate 34 ml/kg actual body weight) and potassium repletion on the first day. 

**Figure 1:**
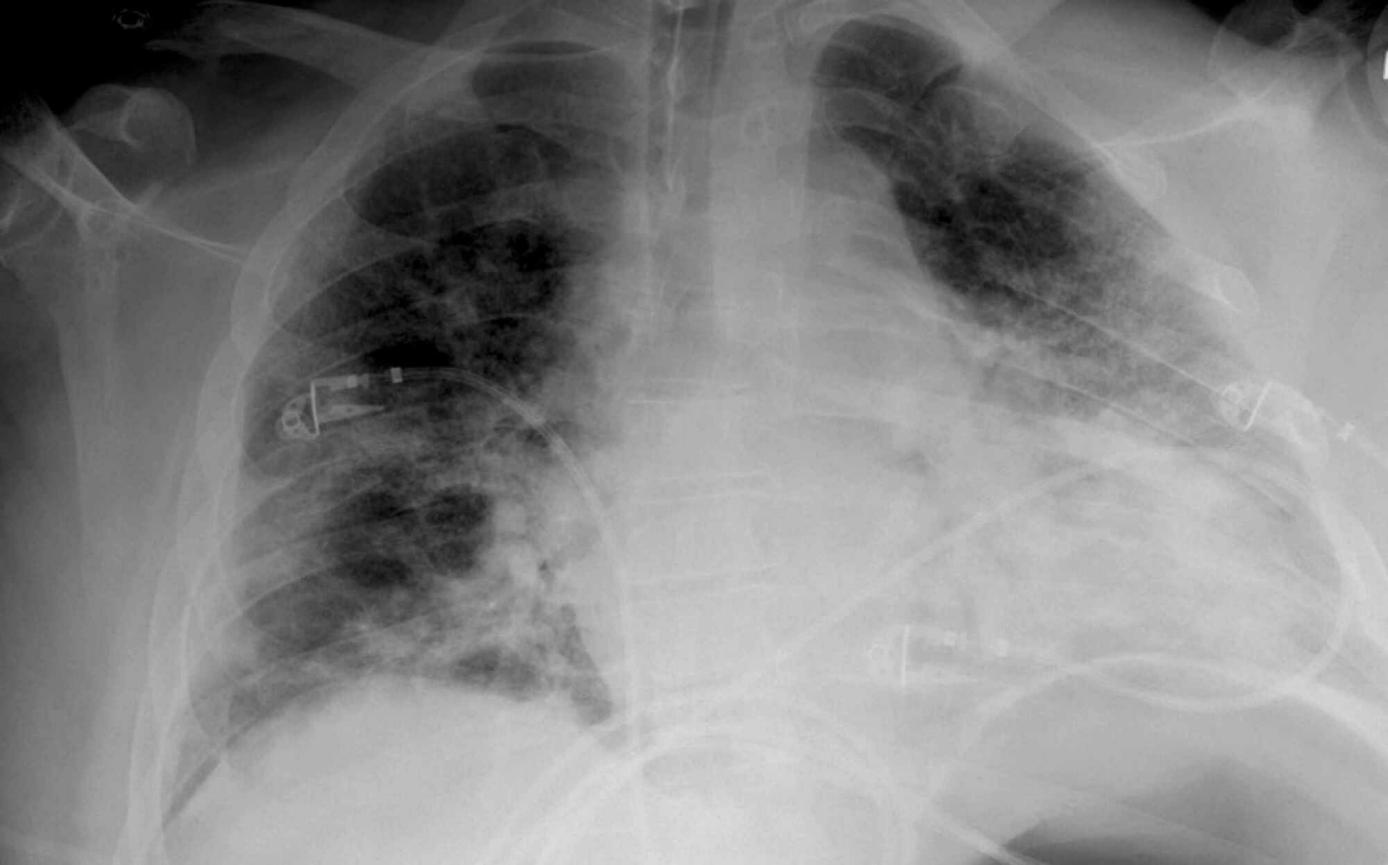
Portable chest x-ray after intubation showed bilateral patchy pulmonary opacities in the mid and lower lung zones without interstitial edema.

On the same day, his hypoxia worsened with an increase in opacities bilaterally on his chest x-ray (Figure 1), so intravenous fluid delivery was ceased and norepinephrine infusion was commenced for 24 hours. 

**Figure 2:**
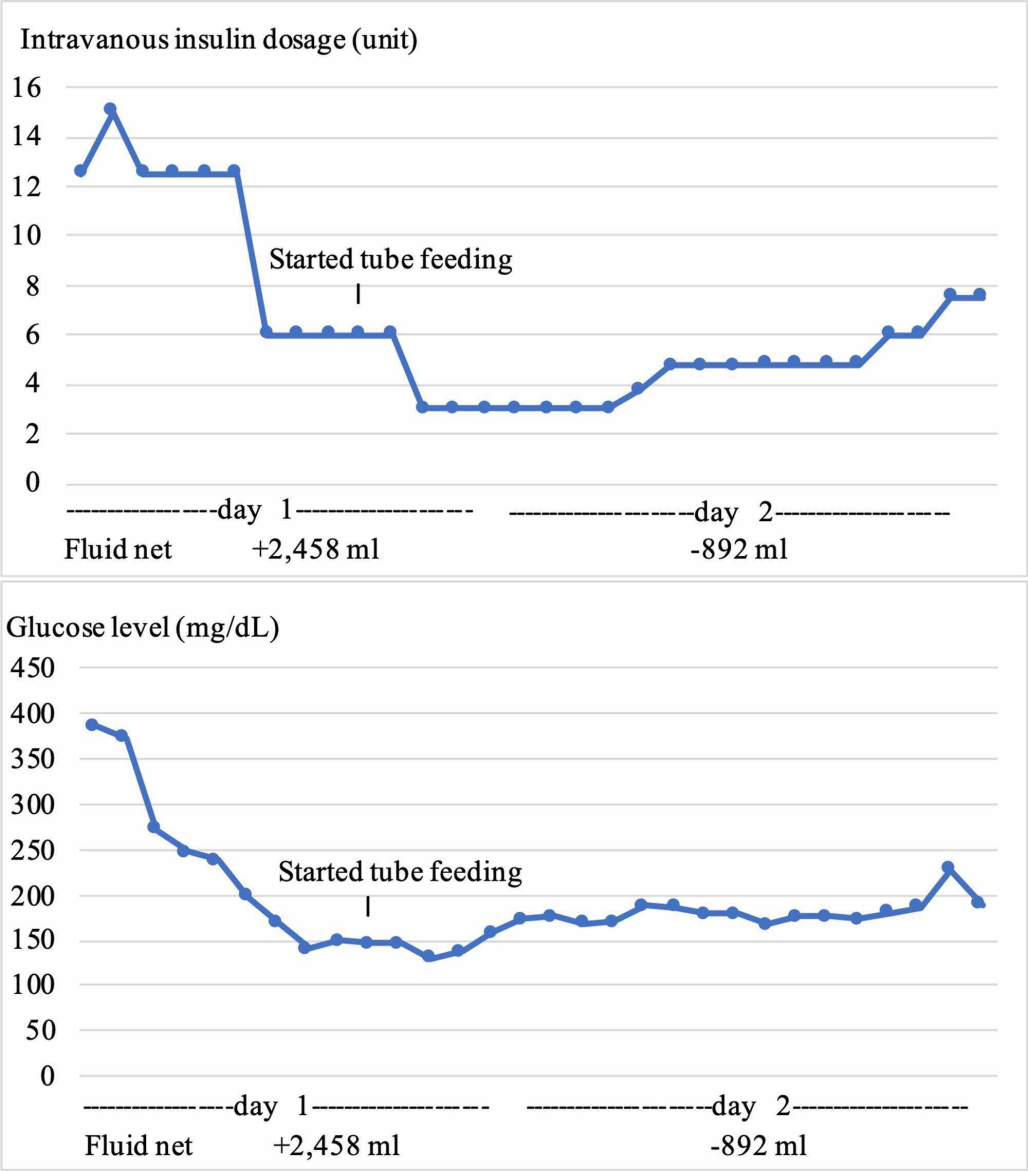
Glucose levels, intravenous insulin dosage, and fluid status during DKA interval. DKA; diabetic ketoacidosis

His mechanical ventilation settings followed ARDS guidelines with low tidal volume (ml/kg ideal body weight). The initial settings comprised of tidal volume 6 ml/kg based on ideal body weight, positive end-expiratory pressure (PEEP) 12 cmH2O, FiO2 75%, with lung compliance of 70 ml/cm H2O, and plateau pressure of 20 cm H2O. Mean PEEP, FiO2, lung compliance, and plateau pressure throughout mechanically ventilated period were 8.5 cmH2O, 60.5%, 55.3 ml/cmH2O, and 18.33 cmH2O, respectively. The patient did not require any prone positioning or neuromuscular blockade. After maintaining a negative fluid balance with volume restriction and furosemide administration, we were able to wean down on PEEP and extubate in 72 hours. However, with continuation of negative fluid balance, he required an additional five days to completely wean off oxygen. Intravenous insulin was continued for 46 hours then was switched to subcutaneous basal-bolus regimen (Figure 2). The patient was admitted for a total of 10 days, three of which he spent in the ICU. He was discharged with insulin degludec 100 units with insulin lispro sliding scale, metformin, and sitagliptin. Dulaglutide was held due to duplication of therapy.

Infectious workup revealed positive COVID-19 from real-time reverse transcription-polymerase chain reaction. Blood culture and sputum culture were negative. The patient was treated with intravenous ceftriaxone, azithromycin, and oral oseltamivir for five days. There were no confirmed cases in his intermediate care facility before or after his discharge.

## Discussion

DKA is a complication commonly seen in type 1 diabetes, whereas patients with type 2 diabetes are rarely affected. However, with an increase in the number of hospitalizations in DKA over the years, up to 27% of cases were found to be from type 2 diabetes [4]. The major clinical difference between these two types of diabetes is a lesser degree of acidosis and absence of hyperkalemia in type 2 diabetes [5].

There are reports of ARDS as a serious complication in severe DKA in adults and children [6,7], yet no data for concomitant DKA and ARDS has been published. All of those patients presented with severe acidosis and hypotension and were managed with aggressive volume resuscitation and insulin infusion. Inflammation and altered vascular permeability were postulated as causes of ARDS [6,7]. Even though our patient presented with ketoacidosis and hypotension, the degree of acidosis was milder compared to those reports. It is unlikely in our case that ARDS was a consequence of DKA. 

Infection is a common cause of DKA in diabetic patients. Published data from 658 hospitalized patients with COVID-19 showed 20% of diabetics suffered from DKA [8]. Apart from the infectious process, treatment with corticosteroids in critically ill COVID-19 patients can aggravate hyperglycemia [9]. Infected patients were also prone to develop ketosis regardless of diabetes status with a prevalence of 7% [8]. Patients with diabetic ketosis tend to have a longer length of hospital stay (33.0 days vs 17.0 days, p=0.003) and lower serum bicarbonate (22.9 vs 26.0, p=0.012) than patients without diabetes, but complications like ARDS (40.0% vs 22.2%, p=0.387) and mortality rate (33.3% vs 14.8%, p=0.131) were insignificantly different [8]. Our patient was extubated within 72 hours and discharged within 10 days, which is shorter than the average length of stay reported in the studies [8,10].

Our patient demonstrated a risk of severe COVID-19 infection due to his underlying conditions, including diabetes and obesity. Diabetic patients with obesity are known to have impaired immunity due to their chronic inflammatory state [2]. Moreover, COVID-19 infection could aggravate their insulin resistance [2]. The risk of severe DKA might be higher based on the studies in severe acute respiratory syndrome (SARS) patients as the virus causes damage to beta islet cells in the pancreas [2,11], though a well-defined mechanism is still unknown. The management of DKA in COVID-19 infected patients involves intravenous insulin therapy, electrolyte replacement, especially potassium, as hypokalemia is frequently seen with this infection, and cautious fluid resuscitation due to potential of worsening pulmonary edema in patients with inflammatory lung [2].

The respiratory system is the main target organ for COVID-19 due to abundance of angiotensin-converting enzyme-2 (ACE-2) receptors that were discovered in alveolar type 2 cells and other respiratory epithelium [12]. Lung injury and ARDS in COVID-19 are associated with excessive immune response including innate and adaptive immunity, cytokine release and immune complex cascade [9]. Inflammatory cytokines, especially interleukin 1 (IL-1), interleukin 6 (IL-6), and interferon gamma (IFNγ) were found in patients with pulmonary inflammation and ARDS [9]. Risk for development of ARDS was increased among elderly (≥65 years), diabetics, patients with neutrophilia, high LDH, and elevated ferritin level [13].

In ARDS, a decrease in surfactant production and altered vascular permeability leads to the accumulation of exudates in alveoli resulting in impaired gas exchange and lung compliance. Excessive fluid volume can worsen oxygenation due to elevation of pulmonary artery wedge pressure. On the other hand, intravascular volume depletion from DKA can cause shunt effect in the lung which can aggravate hypoxia [14]. Therefore, finding optimal fluid balance is essential. A multicenter randomized controlled trial has shown better outcomes between conservative and liberal fluid resuscitation in patients with acute lung injury. A decrease in duration of intubation and length of ICU stay was observed in the conservative group, however, the mortality rate was indifferent [15].

A conservative fluid resuscitation is also crucial in the management of the DKA patient with congestive heart failure or end-stage renal disease requiring hemodialysis. These patients are very sensitive to volume resuscitation, hence require an attentive assessment of volume status [16]. Minimal fluid bolus, i.e. 250-500 ml is recommended in hypovolemic patients with subsequent monitoring for signs/symptoms of volume overload. Intravenous insulin is the principal treatment to improve acidosis by preventing ketone production [17]. It is preferred over subcutaneous insulin due to shorter half-life and initial rapid reduction of glucose level [18].

Currently there are no definite guidelines for DKA and ARDS, hence we managed the patient according to available recommendations [17,19], ventilating with low tidal volume and high PEEP. Intravenous diuresis and vasopressors via central venous access were used to restrict volume replacement and maintain optimum mean arterial pressure. Intravenous insulin was used as the main therapy in volume-restricted patients as observed in DKA with heart failure [20] or with euvolemic dialysis-dependent end-stage renal disease [17]. This case of DKA combined with COVID-19 with ARDS demonstrates another challenge related to competing strategies to achieve optimal fluid status in the era of COVID-19.

## Conclusions

Fluid resuscitation in DKA is imperative, however, with COVID-19-associated ARDS, conservative volume management and vasopressors must be carefully balanced along with intravenous insulin. We propose that the management of DKA in COVID-19 patients with ARDS may be similar to the paradigm utilized for other volume restriction in patients with congestive heart failure and end-stage renal failure. 
